# The full spectrum of climate change adaptation: testing an analytical framework in Tyrolean mountain agriculture (Austria)

**DOI:** 10.1186/s40064-016-3542-1

**Published:** 2016-10-22

**Authors:** Heidelinde Grüneis, Marianne Penker, Karl-Michael Höferl

**Affiliations:** 1 Institute for Sustainable Economic Development, University of Natural Resources and Life Sciences, Vienna, Austria; 2 alpS GmbH, Innsbruck, Austria; 3 Institute of Geography, University of Innsbruck, Innsbruck, Austria

**Keywords:** Climate change adaptation, Regional adaptation, Autonomous adaptation, Mountain agriculture

## Abstract

Our scientific view on climate change adaptation (CCA) is unsatisfying in many ways: It is often dominated by a modernistic perspective of planned pro-active adaptation, with a selective focus on measures directly responding to climate change impacts and thus it is far from real-life conditions of those who are actually affected by climate change. Farmers have to simultaneously adapt to multiple changes. Therefore, also empirical climate change adaptation research needs a more integrative perspective on real-life climate change adaptations. This also has to consider “hidden” adaptations, which are not explicitly and directly motivated by CCA but actually contribute to the sector’s adaptability to climate change. The aim of the present study is to develop and test an analytic framework that contributes to a broader understanding of CCA and to bridge the gap between scientific expertise and practical action. The framework distinguishes three types of CCA according to their climate related motivations: explicit adaptations, multi-purpose adaptations, and hidden adaptations. Although agriculture is among the sectors that are most affected by climate change, results from the case study of Tyrolean mountain agriculture show that climate change is ranked behind other more pressing “real-life-challenges” such as changing agricultural policies or market conditions. We identified numerous hidden adaptations which make a valuable contribution when dealing with climate change impacts. We conclude that these hidden adaptations have not only to be considered to get an integrative und more realistic view on CCA; they also provide a great opportunity for linking adaptation strategies to farmers’ realities.

## Background

Because of the observability of impacts of climate change (CC) in ecosystems, societies and economies (IPCC 2014), attention to adaptation has increased significantly for the past few years. Numerous scholars analyzed climate change adaptation (CCA) and literature grew at a rate of several hundred papers per year (Perry 2015). Although the concept of CCA has got so much attention from scholars all over the world, the limits and barriers in practice are still manifold (e.g. Adger et al. 2007; Dow et al. 2013; Ford and King 2015). Identifying such obstacles (e.g. Biesbroek et al. 2013; Moser et al. 2010) may be a promising approach to overcome them, but we could also question the role of our conceptualization of CCA and if it actually helps in overcoming such limits and barriers. CCA is seen as a *“process of adjustment”* (IPCC 2014) in response to climatic stimuli (Smit et al. 2000), but this often used definition reflects the “first-generation adaptation” approach that aims at particular adaptation solutions to particular CC problems (Boyd and Cornforth 2013). “Second-generation adaptation” offers a more comprehensive view as it also addresses the context in which hazards occur (Burton et al. 2002) and frames adaptation more in the sense of sustainability and resilience (e.g. Boyd et al. 2008; Ensor 2011; Eriksen and O’Brien 2007). Following an intentional perspective, a very common conceptual differentiation is that between autonomous and planned adaptation (for a more detailed description of different concepts see “Climate change impacts and mountain agriculture” section). Autonomous adaptation is seen as *“spontaneous adjustment”* (Carter et al. 1994), which *“does not constitute a conscious response to climatic stimuli”* (Malik et al. 2010). Whereas planned adaptations are “based on an awareness” (Malik et al. 2010) and *“require conscious intervention”* (Fankhauser et al. 1999). Although both types are recognized by several scholars (e.g. Smit et al. 2000), most empirical studies focus on planned adaptations (e.g. Füssel 2007; Kates et al. 2012; Moser 2009). However, adaptation in practice does not always follow the concept of planned adaptation, it is often reactive (Adger et al. 2005) and non-climatic stressors play a role for adaptability (McDowell et al. 2014).

Many scholars conceptualize adaptation processes as sequences of “ideal” adaptation phases, which usually start with problem definition, followed by planning processes, such as identification, prioritization and selection of actions, implementation and finally monitoring and evaluation (e.g. Cross et al. 2012; Mirfenderesk and Corkill 2009; Wheaton and Maciver 1999). This concept of planned adaptation focuses on climate change as key driver for adaptation and leaves hardly any room for other drivers although interactions between climate change and other driving forces, such as demographic change, globalization or social polarization are plain to see (Simonet and Fatoric 2015).

Against this background, we want to provide an analytic framework for a functional perspective on adaptation that considers all actions—intentional, semi-intentional and non-intentional—that improve a sector’s adaption to climate change. A case study from mountain agriculture in Tyrol (Austria) illustrates the applicability of the analytic framework. We selected the case, because mountains are among the most vulnerable regions in the world (Messerli and Ives 1997) and experience climate change impacts earlier and in a more pronounced manner (Ingold et al. 2010). Furthermore agriculture is a sector particularly affected by climate change. Agricultural production primarily takes place outdoors and directly depends on climatic conditions (Lobell et al. 2008; Vermeulen et al. 2012). Adaptation research in mountain regions has a strong focus on developing countries, especially Peru, Nepal and India (McDowell et al. 2014). Studies about mountain regions in ‘developed’ countries are the exception and often focus on tourism (e.g. Behringer et al. 2000; Hoffmann et al. 2009; Scott et al. 2007). CCA is also important to sustain sensitive mountain ecosystems in ‘developed countries’ and their multifunctional services going far beyond food production, such as water provision, biodiversity, landscape amenities, recreation or identity (Schermer and Kirchengast 2006).

With this paper, we aim to offer an analytic framework for an enhanced and extended CCA approach. After summarizing the main climate change impacts in mountain agriculture and CCA concepts, we present the “Study area and methods” section. In a next step, we discuss CC as one of several drivers in mountain agriculture. Then, we present the analytic framework that acknowledges hidden adaptations. We use the case study to illustrate the framework’s applicability. From the case study application, we derive some general conclusion on the conceptualization and empirical analysis of CCA.

## Climate change and adaptation in mountain agriculture

### Climate change impacts and mountain agriculture

CC impacts on mountain agriculture in Austria are expected to be quite ambivalent. On the one hand, mountain regions are among the most vulnerable regions worldwide and agriculture is heavily exposed to climatic conditions and climatic change due to its mode of production (Behringer et al. 2000). Climate variations affect crop yields and lead to changes in food prices (Rosenzweig and Perry 1994), so that several adaptation options could be identified, such as irrigation or changing cropping practices, patterns, and planting dates (IPCC 2014). This might result in the assumption that mountain agriculture is very vulnerable and there is a high demand for adaptation. On the other hand, mountain regions in Austria are often seen as a “winner” of climate change. A general raise in crop and pasture yields is predicted for mid- to high-latitude regions in case of moderate warming (IPCC 2014). Mitter et al. (2014) predict a strong growth in productivity of grasslands, which is the main land use type in Tyrol until the middle of the twentyfirst century. Observations show that the temperature rise from nineteenth century until the end of the twentieth century in the Austrian Alps was about twice as large as the northern-hemispheric average (Auer et al. 2007). However, climate change effects include direct and indirect effects that go beyond increases of temperature and CO_2_ concentration, which are mainly considered as responsible for the productivity growth.

**Fig. 1:**
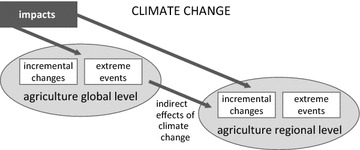
CC impacts on mountain agriculture on a regional level

The following categorization shows three different CC effects on mountain agriculture (see Fig. 1). The first shows impacts (incremental changes and extreme events) on a global level. These impacts can change global framework conditions which, in turn, do also indirectly affect the regional level. The other two do directly affect the regional level, but are very different in their frequency of occurrence and intensity: incremental changes and extreme events.

#### Incremental changes

These steady changes (mainly in temperature and precipitation) were observed in the past and can be predicted for the future. In Austria, the average temperature has increased by two degrees since 1880 (global: one degree) and is expected to further increase by 1.4 degrees until 2050 (APCC 2014). An increase in precipitation of 10–15 % was observed in mountainous western Austria during the last 150 years. In the twentyfirst century an increase in precipitation is expected for the winter season, while a decrease can be expected for the summer season. The extent of change in precipitation varies between different models (Gobiet et al. 2014). Neither can we see a clear trend for the annual average precipitation (APCC 2014). A decrease in the duration of snow cover as well as a decline in surface and volume of all Austrian glaciers has been observed in recent decades (APCC 2014). These incremental changes have direct impacts on mountain agriculture. The growth in productivity on grasslands due to temperature rise and higher CO_2_ concentration in western Austria has already been highlighted above (Mitter et al. 2014). Higher temperatures may also favor the spread of invasive weeds or harmful organisms and lead to quality losses of agricultural products (IPCC 2014). Rising temperature can also become an important limiting factor for livestock, as productivity may be reduced and the risk of disease increases (APCC 2014, IPCC 2014).

#### Extreme weather events

Extreme weather events, such as storms, hail, heavy rainfall or droughts, characteristically occur very irregularly and lead to immense damage. Impacts of extreme events on agricultural production are difficult to quantify with models, as they cannot be adequately calibrated and tested (IPCC 2014). Temperature extremes have changed remarkably and this development will continue in the twentyfirst century and lead to more heat waves (APCC 2014). There are no consistent trends for heavy rainfalls, but models usually show an increase of heavy rainfalls from autumn to spring (APCC 2014). Despite some outstanding storm-events during the last few years, a long-term rise in storms cannot be proved (APCC 2014). Due to the high degree of uncertainty, useful statements regarding the development of extreme weather events can only be made with caution. Extreme weather events however gravely affect agriculture via erosion, harvest shortfalls or damage to farm buildings and infrastructures. Economic effects of extreme events increased during the last three decades in Austria (APCC 2014).

#### Global effects of climate change

Climate change also impacts on global food markets, which then indirectly affect the regional level. Global agricultural production and markets will be strongly affected by a growing scarcity of water, fluctuating yields, growing transport costs, increase in land-use conflicts and growing costs of production inputs, such as energy, fertilizer and water (BMLFUW 2012). Global agricultural yields are expected to significantly decline by the middle of this century (IPCC 2014). Observations of past climate extremes in key production regions showed rapid food and cereal price increases (IPCC 2014). Extreme climate and weather events will very likely further reduce food production (IPCC 2014).

### Conceptualizing climate change adaptation

The latest IPCC report defines CCA *as “the process of adjustment to actual or expected climate and its effects. In human systems, adaptation seeks to moderate or avoid harm or exploit beneficial opportunities. In some natural systems, human intervention may facilitate adjustment to expected climate and its effects”* (IPCC 2014). Just like this IPCC characterization, most CCA definitions refer to adjustment in a system in response to climatic stimuli (Smit et al. 2000). Some scholars distinguish different forms of CCA by most commonly referring to conceptual differentiations like reactive versus anticipatory or autonomous/spontaneous vs. planned adaptation (cf. Table 1).

**Table 1:** Common conceptual differentiations of climate change adaptation

Authors	Differentiation between		Differentiation between	
	Reactive CCA	Anticipatory CCA	Autonomous/spontaneous CCA	Planned CCA
Fankhauser et al. (1999)	Reactive measures are those that institutions, individuals, plants and animals are likely to make in response to climate change, after the fact	Anticipatory adaptations are deliberate decisions to prepare for potential effects of climate change. They are taken in advance of climate change, before the fact	Natural or spontaneous adjustments in the face of a changing climate	Planned adaptation requires conscious intervention
Malik et al. (2010)	Adaptation that takes place after impacts of climate change have been observed	Adaptation that takes place before impacts of climate change are observed. Also referred to as proactive adaptation	Adaptation that does not constitute a conscious response to climatic stimuli, but is triggered by ecological changes in natural systems and by market or welfare changes in human systems	Adaptation that is the result of a deliberative policy decision, based on an awareness that conditions have changed or are about to change and that action is required to return to, to maintain, or to achieve a desired state
Smit et al. (2000)			Adaptations which occur in systems as a matter of course	Those that require or result from deliberate “policy decisions”. Planned adaptations may be distinguished by the intent and timing of the initiative
Füssel (2007)	…after some impacts have been experienced			Planned adaptation means the use of information about present and future cc to review the suitability of current and planned practices, policies, and infrastructure

Beside these two very common conceptual differentiations, numerous others can be found. Smit et al. (2000) for example introduced an adaptation typology, which focuses on (1) timing relative to stimulus, (2) intent (autonomous vs. planned), (3) spatial scope, (4) form and (5) degree of necessary change. Eakin et al. (2014) identify three different adaptation approaches: (1) social vulnerability approaches, (2) resilience approaches and (3) targeted adaptation approaches while Castán and Bulkeley (2013) focus on a governance perspective (Castán and Bulkeley 2013). Biagini et al. (2014) provide a comprehensive overview of existing adaptation typologies and developed a new typology which includes capacity building, policy, information, technology, etc.

Although spontaneous adaptation is receiving some attention within these conceptual differentiations (e.g. Adger 2001; Thorn et al. 2015), most scholars remain in the tradition of an intentional perspective on CCA and consequently focus on planned, impact-driven and anticipatory adaptation (e.g. Kates et al. 2012; Marshall et al. 2014; Moser 2009). Starting with Burton et al. (2002) several limitation of this “first generation” CCA have been discussed (Dessai and Hulme 2004; O’Brien et al. 2004). One point of critique focuses on the belief that planned, anticipatory adaptation has to be created from scratch, fading out existing economic, social and/or political driving forces and constraints acting on a sector’s actors. Becoming more aware of this ‘polyrational context’ in which adaptation occurs, opens up the possibility, that also non-climatic driving forces can trigger interventions that directly or indirectly influence the coping capacities of actors to whatever changes the future might bring. Burton et al. referred to such interventions as adaptation policies that already exist, but are “rarely recognized by that name” (Burton et al. 2002). Thus, trying to ground adaptation in such polyrational contexts inspired the discussion about “second generation” adaptation assessments, reframing adaptation as managing for resilience, not impacts (Boyd and Cornforth 2013). As Darnhofer (2010) pointed out, resilience thinking could be helpful in enhancing adaptive capacity. The resilience perspective is not yet reflected in the concepts presented in Table 2.

**Table 2:** Overview of CCA actions in Tyrolean mountain agriculture

No.	CCA action	Connection to CCA	No.	CCA action	Connection to CCA
1	Extended mountain grazing	Extension of the vegetation period requires adaptation of the grazing management	16	Gene bank of the province of Tyrol	See 9
2	Drought tolerant varieties for grasslands	Droughts require tolerant varieties to minimize losses	17	Initiative for climate protection and domestic food	Raising awareness for regional food helps to strengthen “climate-adaptive-friendly” products
3	Hail-nets	Hail-nets protect sensitive crops from hail damages	18	“Tiroggl”—regional rye bread	See 2
4	Low-input systems	Changing global conditions lead to price volatilities. Thus, farms which are more independent of external resources (e.g. fodder) are more resilient to the impacts of CC	19	“Fisser Gerste”—regional barley cultivation and marketing	Ancient grains have a broad diversity of genetic resources, which can contribute to adaptation
5	Organic farming	Organic farming is better adapted to climate change in some aspects (e.g. humus content of the soil)	20	“LEBA” association for information, consultation and marketing of regional, organic products	Organic farming is better adapted to climate change in some aspects. Moreover, awareness raising of customers towards “climate-adaptive friendly” products is essential to create a demand for such products
6	“Schmatzi”—awareness raising for young children	Raising awareness for domestic food among the youngest helps to strengthen “climate-adaptive-friendly” products	21	Tyrolean mountain pasture pig	One CCA measure is to preserve mountain pastures. Developing innovative products that achieve an added value from the pastures can make an important contribution
7	“School on the farm”	Raising awareness for domestic food among children helps to strengthen “climate-adaptive-friendly” products	22	Quality Tyrol—direct marketing of domestic products	Creating regional sales opportunities reduces farmers’ dependencies on retail chains and exports. This makes the farmers more resilient when global conditions change
8	Sustainability prize	Sustainable producing farms are in many aspects better adapted to CC. Hence, raising awareness in this field is fundamental	23	Project: “Almleben”—strengthening added values and economic performances on mountain pastures	See 22
9	Project: CereAlps—collection, and preservation of landraces	Ancient grains have a broad diversity of genetic resources, which can contribute to adaptation	24	“Bio vom Berg”—producer-owned brand for organic products from Tyrol	Organic farming is better adapted to climate change in some aspects (e.g. humus content of the soil)
10	Ancient Tyrolean grains	See 9	25	“Gutes vom Bauernhof”—direct marketing of domestic products	See 22
11	Project: Gene-Save - potential uses of bread grain landraces	See 9	26	Association for defense and research of hail	Although the link between climate change and an increase of hail is not scientifically evident, damage sums are rising and hail defense can be an adaptation strategy
12	Project: Organic mountain agriculture in Tyrol	Direct connection to CCA within the project	27	Insurances against natural disasters (droughts in pastures)	Insurance covers for extreme events, such as droughts, help farmers to overcome serious damages
13	Project: Capital Adapt—the role of social and human capital regarding climate change adaptation	Direct connection to CCA within the project	28	Association of farm women	The farm women’s network strengthens social and human capital which enhance resilience against CC (esp. extremes)
14	Project: Clim Grass—climate change impacts in grasslands	Direct connection to CCA within the project	29	“Quality of life on farms”	Strengthening farmers personal resources enhances resilience against CC
15	Tyrolean climate strategy	Direct connection to CCA within the strategy			

To provide a poly-rational framework for CCA that includes the resilience perspective, we propose a threefold concept of climate change adaptation, ranging from “explicit” (intentional) to “hidden” (non-intentional) interventions. The latter one, we define as strategies, activities, initiatives that contribute to a sector’s (or a city’s, or a region’s) adaptability to climate change, although being motivated by driving forces that do not directly relate to climate change.

## CCA: a comprehensive approach

Although CCA received so much attention from academic scholars, we cannot automatically assume this is also reflecting in farmers’ discourses. Several drivers steer agricultural production and marketing and many of them are much closer to farmers’ perceived priorities than climate change. However, CCA literature usually focuses on narrow conceptualizations of CCA (see “Conceptualizing climate change adaptation” section) that basically ignore other constraints and drivers of the sector.

### Explicit and hidden CCA

In addition to obvious, clearly labelled CCA efforts, there are actions which do not seem to have any connection to CC at first sight. They are motivated by economic, ecologic, social or other drivers, but as side-effects they might also contribute to CCA. According to the common definitions of CCA (see “Conceptualizing climate change adaptation” section), such actions are not conceptualized as CCA at all, particularly, if the measure’s contribution to CCA is not obvious. This brings us back to the question of what CCA is. These adaptations “by accident” are not part of common CCA concepts, as described above. But such actions may raise the sector’s resilience and contribute to its adaptability to climate change. Such hidden adaptations overlap with the category of autonomous/spontaneous adaptation (see Table 2). However, the consideration of purposeful, planned adaptation to other drivers of the sector is not represented in any common CCA differentiation. Whereas conventional conceptualization of CCA assume intentional adaptations to climate change (planned and pro-active CCA) or the lack of any intention (spontaneous/autonomous adaptation), hidden adaptations focus on adaptive measures that are motivated by other, non-CC drivers. In the following sub-section, we propose an analytic framework for an integrative empirical analysis of all activities that can be considered as contributing to the sector’s adaptability for future climate change impacts.

### The analytic framework acknowledging hidden climate change adaptation

**Fig. 2:**
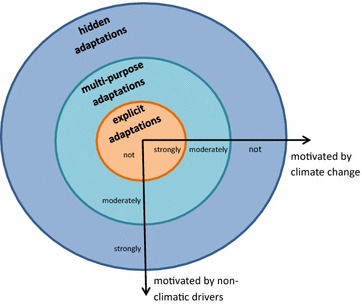
Differentiation of CCA actions according to motivation

The following framework should support not only the analysis of planned and anticipatory CCA, but also the identification of all actions that can contribute to CCA. This model divides CCA actions into three different categories: explicit, multi-purpose and hidden CCA actions (see Fig. 2). The basic idea is to distinguish between the (main) intentions motivating every action. In general, the influence of climate change as motivator for adaptation decreases from explicit to hidden actions (explicit CCA—strong influence, hidden CCA—weak/no influence). The other way round, the influence of non-climatic drivers increases from explicit to hidden actions (see Fig. 2). Explicit actions are typical CCA measures, which are initiated in direct response to climate change and which are addressed by common CCA definitions. There is no doubt that these are CCA efforts since they are usually also labelled as CCA. Besides climate change, other drivers, such as changing prices or policies influence multi-purpose actions. In contrast to explicit CCA, these actions are not only driven by climate change, although the change of climatic conditions may be of great importance for the initiators. The CCA labelling varies—some are clearly labelled as CCA, while others are not. Hidden actions are only motivated by non-CC drivers, such as policies, markets, or life-style changes. CCA was not an intention, but occurs as an unplanned side-effect. Of course, these actions are not labelled as CCA. Although these actions do not fit within common CCA definitions, they can significantly contribute to an agricultural sector well adaptable to climate change.

Of course, the transitions between the three categories are fluid and a clear assignment is sometimes difficult. The main purpose of this analytic framework is to support the empirical analysis of actions contributing to CCA, although they were motivated by other drivers. This integrative analysis is important to include all different types of measures contributing to CCA. Furthermore, those actions that were motivated by other drivers hold potential for learning on how CCA strategies can be better tied up to farmers’ everyday life. The framework can be extended to include the different effects of climate change: incremental changes, extreme weather events and global effects of CC (see Fig. 4). This further differentiation can also focus on different policy level, different sectors or actors. The differentiation in CCA degrees and CC effects (as shown in Fig. 4) helps identifying CCA gaps and to check if all sectors, impacts etc. are targeted or if the allocation of activities to different organizations or policy levels is unbalanced. We apply the analytic framework to the practical example of Tyrolean mountain agriculture to test its applicability and usefulness.

## Study area and methods

### The study area of Tyrol

We selected Tyrolean agriculture as a typical case for many other mountain regions in the European Alps characterized by milk production (e.g. in Switzerland, Bavaria). Tyrolean agriculture is small-scaled and has a strong focus on milk production with permanent pastures. Tyrolean farms are registered in the national land register of mountain farms (“Berghöfekataster”), which qualifies them for EU co-financed compensatory payments for difficult production conditions in less-favored areas. Additionally, farmers have access to financial support from other agricultural policies, such as the Austrian agri-environmental scheme—so that on average 22 % of the farm income is from public funds (Land Tirol 2014).

In addition to food production, Tyrolean farmers sustain cultural landscapes, which are important for tourism, protection against natural risks, regional identity as well as bio-cultural diversity (Schermer and Kirchengast 2006). Agricultural land in Tyrol is under pressure as only 11 % of the total surface area is appropriate for settlement and agricultural production. Furthermore, one quarter of the total area is under some sort of nature protection. Thus, there are various conflicting from settlement, tourism, agriculture, forestry, or conservation on a relatively small area. Today, only 3 % of the working population are farmers (1961: 24.9 %, 1971: 11.4 %), and only 28.5 % work as full-time farmers (Statistik Austria 2014). Organic agriculture plays an important role (23.28 % of farmed land).

### Data collection and analysis

Between June 2014 and June 2015, qualitative data were collected through 20 semi-structured key-informant interviews with representatives from core agricultural sector organizations, such as the Chamber of Agriculture, the Austrian Federal State of Tyrol, the Association of Farm Women, the Association of Organic Farmers, the main agricultural insurance company (Hagelversicherung) and the Federal Institute for Less Favoured and Mountainous Areas. These interviewees were selected due to their insights into the sector and its governance. It was assumed that they are familiar with governmental strategies and regulations as well as with actions in practice. The interviews were organized in four parts: within the first—introduction—the interviewees were asked about awareness and significance of climate change within their organizations and within agriculture in general. The second part focused on their own CCA awareness, which might go beyond that of their organization. Within the third part, interviewees were asked about initiatives and actions relevant for CCA and the last part focused on further organizations and persons, which might be active in the field of CCA. The data obtained were complemented with interviews with academic experts specialized on mountain agriculture. All interviews were recorded and transcribed. Results of the interviews were supplemented with desktop-research of relevant documents on the Tyrolean agriculture.

The aim was to investigate climate change adaptation initiatives in the Tyrol’s agricultural sector and in associated regional organizations. Therefore, we did not include higher level adaptation strategies on the European or national level (e.g., the national agri-environmental scheme). We transcribed and paraphrased the interviews according to qualitative content analysis (Mayring 2010). The analysis focused on CCA actions characterized by at least one the following criteria derived from CCA literature: (1) response to climatic stimuli (Smit et al. 2000), (2) increase in resilience (Adger et al. 2005), (3) awareness raising (BMLFUW 2012), (4) research in the field of CCA and (5) organic farming initiatives (Borron 2006, Niggli et al. 2007). The identified actions where categorized into explicit, multi-purpose and hidden CCA actions addressing either extreme weather events, incremental changes or indirect global CCA effects (see Fig. 4).

This procedure of identifying and classifying CCA action that we described above, could easily be adapted for application in other regions or for other sectors. The main efforts involve time for interviews, their transcription and analysis.

## Results from Tyrolean mountain agriculture

### Climate change as one of many challenges

**Fig. 3:**
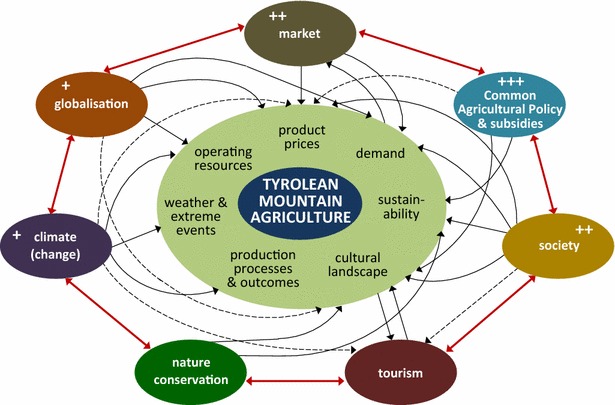
Drivers of change in Tyrolean mountain agriculture (+++, ++, +indicate the relevance of drivers as perceived by interviewees, drivers without any + are of minor concern, full and *dotted lines* show direct and indirect influences as pointed out in the interviews)

Results from the case study show that the great amount of scientific articles and policy strategies for CCA have not yet tickled down to the sector’s practices. Interviewees point out that farmers and their supportive networks are much concerned with more pressing challenges than climate change. As they have to tackle market or policy changes affecting them today and tomorrow, they cannot allocate much time for considering the long-term impacts of climate change. Figure 3 gives an overview of the most important drivers in Tyrolean agriculture as perceived by interviewees. They are inter-connected and interviewees see climate change—if at all—as one of several future challenges in this greater context.

Based on the statements of their representatives, farmers are aware of climate change and do already observe impacts as they live in close contact with nature. Several remarkable extreme events effected agriculture in Tyrol in the last years: droughts in 2003 and 2011 as well as floods in 2005 and 2013. Such extremes raised awareness for climate change. Mass media are the first source of information for most of the farmers, who however do not consider this information as useful for their specific needs. Farmers would need sector specific information and support in dealing with (future) impacts and uncertainties. Furthermore, interviewees emphasize the lack of context-specific recommendations for CCA actions that tie up to farmer’s real world issues. Another obstacle is the lack of well-prepared data that is useful and easily accessible for farmers. The *“Austrian Assessment Report Climate Change”* (APCC 2014) gained some attention among the interviewees. It also addresses agriculture and provides information about specific impacts, effects and measures. In 2012, the Austrian government released a *“Strategy for adaptation to climate change”* (BMLFUW 2012), which is hardly known by the interviewees. Interviewees see the missing networks between science and practice as another obstacle for the implementation of CCA measures.

The interviewees although well aware of the climate change discourse cannot identify any explicit CCA efforts in the Tyrolean agricultural sector, yet can identify several hidden adaptations which they did not associate with climate change. Interviewees of the chamber of agriculture, which is the legal representative body for all farmers and the primary provider for agricultural expertise and farm related administrative services, rank climate change rather as low or moderate priority. One of the representatives interviewed even questioned the existence of anthropogenic climate change. The interviewees point out that forecasts of future climate change impacts are often very fuzzy with much uncertainties, which make it very difficult to come up with concrete CCA measures.

The interviewees have difficulties in identifying explicit CCA efforts, although most interviewees are resigned to the unavoidable fact of CC.

## The application of the analytic framework: hidden adaptation measures in Tyrolean mountain agriculture

Table 2 gives an overview of all CCA actions identified from interviews and document analysis. As pointed out in the “Methods” section, we focused on actions within the Federal Province of Tyrol, which means that national and EU level actions were not considered. Austrian provinces have a relatively broad scope of action regarding CCA since many relevant competences are allocated to the nine regional governments in Austria (e.g. Spatial Planning Law, Nature Protection Law, social policies). Furthermore, the provinces are responsible for the implementation of national and EU policies on agriculture, water or soil protection.

As expected and conceptualized, the connections of the above actions to CCA are often “hidden” and not at all explicit. For example, the association of farm women supports the development of humans and organizations in agriculture and in rural areas in the face of changing production and living conditions. This association strengthens social capital within and social learning among the farmers’ community and therefor enhances adaptation processes and the sector’s resilience. Better connected farmers are better able to learn from each other and to help each other—also in case of extreme weather events. Additionally, knowledge and experiences in dealing with climate change impacts and adaptation can be shared.

**Fig. 4:**
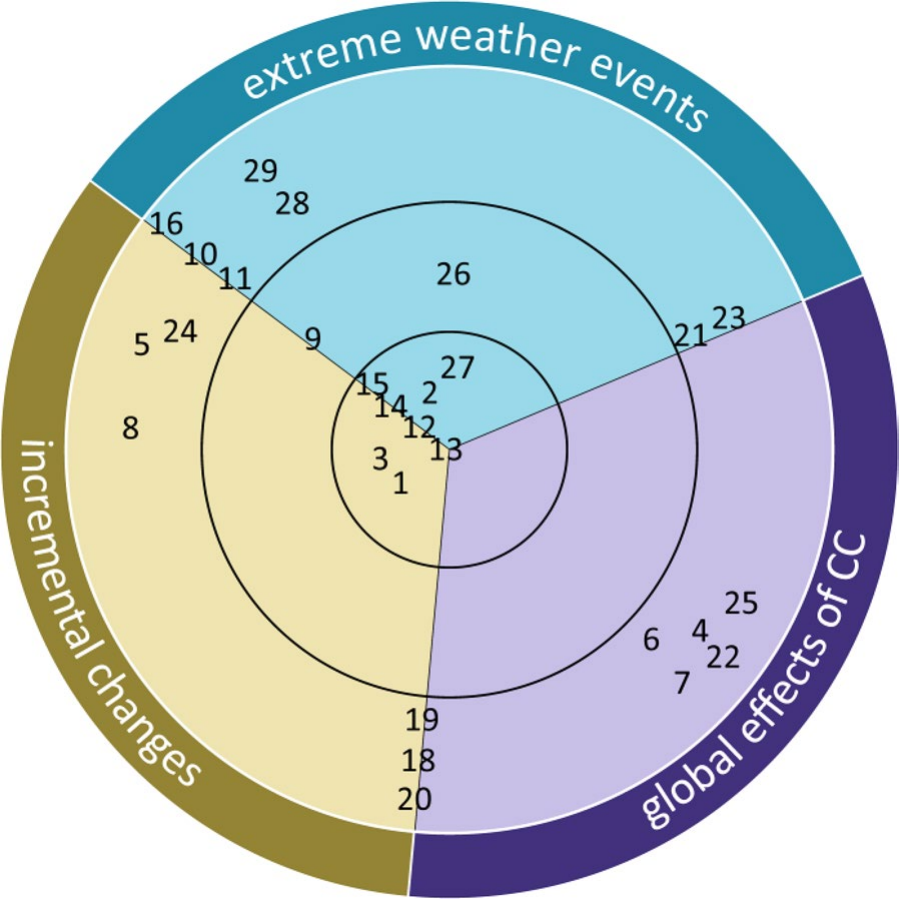
Explicit and hidden CCA actions in Tyrolean mountain agriculture

Figure 4 shows the classification of the CCA actions from Tyrolean mountain agriculture. Some CCA actions can contribute to more than one CC effect; these are located directly on the transition line (e.g. action no. 9, 15, 20). It has to be added, that the classification of the CCA measures to a CCA category and to one of the three CC impacts was not completely straight forward in every case due to some scope for interpretation. The allocation was guided by the objective to provide an overview on all CCA actions identified for the Tyrolean agriculture sector and to identify focal areas as well as neglected areas.

The division into the three categories of CCA shows that eight out of totally 29 measures can be considered as explicit CCA—actions in direct and only response to CC. Only two actions are categorized as multi-purpose CCA and a clear majority of 19 actions belong to hidden CCA. This concentration of hidden CCA deserves particular attention as these actions do not count as CCA in the common view. The second differentiation is relating to the different effects of CC. A clear allocation to one specific field was neither possible nor meaningful for those 14 CCA actions that address two CC effects and are thus located on the transition line between those two. 15 actions were directly allocated to one field of which five were associated with incremental changes. Global effects of CC and extreme weather events got five actions each. It is striking that there are no explicit and even no multi-purpose adaptations which address global CC, which means all eleven CCA relevant actions categorized in the field of global effects of CC (exclusively and on the transition line to the other fields) are hidden adaptations. This shows the exclusion of these effects in planned and anticipatory CCA although some strategies highlight their relevance.

## Discussion and conclusions

In this paper, an analytic framework for a more differentiated and integrative analysis of CCA is proposed, which aims at stimulating a debate on what is CCA and how it can tie up to actors’ realities. This framework was developed in response to the common CCA concept, which seems problematic especially in two points:

1. *Sector*-*specific drivers and constraints are not taken into account*: The common view of CCA puts climate related efforts into the center and basically ignores other sector-specific conditions and drivers. But farmers interact in a complex field of multiple pressures and drivers with various changes they have to adapt to. The perceived priority of these changes varies, but none of the interviewees ranked climate change among the top three priorities. The most important drivers within a sector should be taken into considerations for possible synergies for CCA, as they constitute the realities of those who would be responsible for implementing CCA measures.
2. *Lack in practical orientation*: The majority of all CCA relevant actions identified for the case study of Tyrolean mountain agriculture would not count according to the common CCA definitions. But these hidden actions create adaptability regarding multiple changes and could thus be role models for further CCA actions with higher acceptance and with beneficial effects for the overall adaptability of the sector to whatever the future might bring. In view of high uncertainties of climate change models, this might also be the more reasonable way than the illusion of pro-active and planned adaptation to a future that we cannot fully understand from today’s perspective.

## Results from Tyrolean mountain agriculture

The results from the case study show that regional adaption actions are very broad and occur in various ways. As in other studies (e.g. Comoé and Siegrist 2015), interviewees expect that most Tyrolean farmers are aware and already have observed impacts of climate change. Nonetheless, only a few explicit CCA were identified. More than half of the identified actions were not motivated by climate change. Such hidden or multi-purpose did not gain much recognition in past CCA literature. Even autonomous CCA gained only little attention in literature (Thornton and Manasfi 2010), as most scholars focus on planned CCA (e.g. Füssel 2007; Geneletti and Zardo 2016). Bonzanigo et al. (2014) state *“it is crucial to include an assessment of farmers’ autonomous adaptation into the design and evaluation of rural policy measures.”* It can be assumed that hidden and multi-purpose CCA action do not only play a role in Tyrolean agriculture, but will also be relevant in other sectors and elsewhere. These “hidden CCA” can be considered as autonomous from the perspective of climate change, but might be clearly planed and motivated from other intentions, such as market stabilization or nature conservation. Tompkins et al. (2010) modified an IPCC definition by adding *“whether or not motivated by climate”* for their assessment of adaptation in the UK and thus considered hidden CCA. It is remarkable that the majority of hidden adaptations from the case study promise an increase of the sector’s resilience by strengthening the farmer’s adaptability through decentral, self-organized social networks for autonomous co-learning and experimentation, while many adaptation measures in literature focuses on technical solutions such as climate information systems or irrigation (Smit and Skinner 2002).

The categorization of CCA actions in the Tyrolean agriculture sector highlight according to the analytic framework highlight two key gaps: firstly multi-purpose actions and secondly actions in the field of global effects of climate change. The underrepresentation of multi-purpose actions reflects the current reductionist approaches to CCA which hardly considers sector specific drivers or constraints. It also shows that the idea of mainstreaming adaptation which aims at integrating adaptation into established or on-going policies, development strategies or management plans (Agrawala 2006) is not yet established. Although it is obvious that global climate change will also impact the regional level (IPCC 2014), adaptation actions in this field are rare. In the liberalized Common Agricultural Policy of the EU, farmers are heavily affected by volatile world market prices (both on markets for production inputs as animal feed, energy as well as output markets for their products) and thus vulnerable to global CC, too. In this line of thought, farmers might be more aware of this indirect effects by focusing on policy and market changes. In contrast, the Austrian Strategy for Adaptation discusses the influence of global conditions on agriculture is discussed, but proposes no specific actions.

It may be pointed out that it cannot be totally clarified to what extent the listed actions actually contribute to CCA. As some contexts to CC and adaptation are very broad, the relevance of the listed actions in Table 1 for CCA may not be clear at first sight. There are already some experiences with ex-ante evaluations of CCA measures, but such endeavors are complex as indicators alone are not enough and specific contexts have to be taken into account (Klostermann et al. 2015). A comprehensive evaluation was outside the scope of this analysis, which focused on testing the applicability of the analytic framework based on a first inventory of actions that can be directly or indirectly connected to climate change impacts.

## Usefulness of analytic framework

The presented analytic framework and its underlying methods were applied to the case of Tyrolean agriculture, but experienced researchers could also easily adapt it for application in any other region or for other sectors, such as tourism or forestry. Such an integrative, sector and regional context specific assessment of CCA actions that also takes into account hidden adaptations could be useful for the identification of gaps in already existing adaptation strategies to better focus scarce public resources on those activities with the highest leverage. One of the strengths of the presented framework is its ability to show the full spectrum of the sector’s adaptation practice which includes intentional as well as unintentional adaptations to CC and other drivers. For an application of this approach for other regions and sectors sufficient resources are necessary as well as insights into and sensitivity for the specific geographical and sectoral context. Interview partners should be selected carefully as they have a big influence on the results. The qualitative interviews need interviewers experienced in qualitative research who are able to generate the atmosphere of a trustful, reflective and honest exchange. About 60–90 min per interview plus time for preparation and follow-up processing have to be calculated. Furthermore, transcriptions and analysis of the results are very time-consuming. But such a context-sensitive and integrative CCA assessment provides much needed insights for a more effective and efficient allocation of scarce public resources for CCA.

Effective adaptation actions should be tailored to regional conditions (Reyer et al. 2012), and consider regional actors’ needs, perceptions and motivations to address the limited “intent to implement” as a notable barrier (Öhlmer et al. 1998). Adger and Barnett (2009) state that “adaptive capacity will not necessarily translate into action”, it needs an initial impulse which could be given through motivation.

The concept of hidden CCA can be connected to the concept of second generation adaptation which does not primarily focus on predicted biophysical threats, but considers special contexts and underlying vulnerabilities (Boyd and Cornforth 2013). The idea of mainstreaming adaptation takes a similar direction and aims at integrating adaptation issues into ongoing (policy) processes to consider sector specific contexts (UNEP 2011). The common idea is to see CCA in the bigger context of sector-specific drivers and motivations, which may help to push the sector’s adaptability. Poly-rational multi-purpose adaptations, as also highlighted by the framework, directly consider climate change next to other sector-specific drivers.

The focus on technical solutions and adaptation measures (esp. in agriculture, e.g. Bird et al. 2016; Esteve et al. 2015) also appears to be problematic. We are more and more often challenged with infrequent and unpredictable extreme events which may result in power failure, but technical solutions mostly depend on electrical power. Many of the hidden CCA are “soft” measures, focusing on long term learning processes, self-organization, local knowledge and adaptive governance, which are considered as key for CCA (Adger et al. 2005; Adger 2010; Wolf 2011).

An increased focus on hidden adaptations, supported by the concepts of adaptive governance and resilience could guide the way to agricultural systems with a higher degree of adaptability that is supported by self-organized networks for social-learning, experimentation and exchange of available resources for coping with unexpected situations. Including these aspects into CC scenarios could also improve the scenarios’ validity and create closer links between CC scenarios and the every-day life of their potential implementers and users.

It is hardly possible to quantify the role of hidden adaptations in numbers. However, the Tyrolean case showed that hidden actions, which have been implemented for diverse motivations, do exist whereas explicit CCA action is scarce. It may be assumed that such hidden CCA actions can be found in other regions and in other sectors as well. This article showed that the suggested framework extending the CCA concept to multi-purpose and hidden adaptive practices can support an integrative perspective on CCA that better ties up to farmers’ realities.
